# Antibacterial and Antibiofouling Activities of Carbon Polymerized Dots/Polyurethane and C_60_/Polyurethane Composite Films

**DOI:** 10.3390/jfb15030073

**Published:** 2024-03-17

**Authors:** Zoran M. Marković, Milica D. Budimir Filimonović, Dušan D. Milivojević, Janez Kovač, Biljana M. Todorović Marković

**Affiliations:** 1 Vinča Institute of Nuclear Sciences, National Institute of the Republic of Serbia, University of Belgrade, Mike Alasa 12-14, 11001 Belgrade, Serbia; mickbudimir@gmail.com (M.D.B.F.); dusanm@vinca.rs (D.D.M.); 2 Jozef Stefan Institute, Department of Surface Engineering, Jamova 39, SI-1000 Ljubljana, Slovenia; janez.kovac@ijs.si

**Keywords:** carbon polymerized dots, fullerene C_60_, polyurethane, composite films, electrostatic force microscopy, viscoelastic microscopy, antibacterial, antibiofouling, photodynamic, reactive oxygen species

## Abstract

The cost of treatment of antibiotic-resistant pathogens is on the level of tens of billions of dollars at the moment. It is of special interest to reduce or solve this problem using antimicrobial coatings, especially in hospitals or other healthcare facilities. The bacteria can transfer from medical staff or contaminated surfaces to patients. In this paper, we focused our attention on the antibacterial and antibiofouling activities of two types of photodynamic polyurethane composite films doped with carbon polymerized dots (CPDs) and fullerene C_60_. Detailed atomic force, electrostatic force and viscoelastic microscopy revealed topology, nanoelectrical and nanomechanical properties of used fillers and composites. A relationship between the electronic structure of the nanocarbon fillers and the antibacterial and antibiofouling activities of the composites was established. Thorough spectroscopic analysis of reactive oxygen species (ROS) generation was conducted for both composite films, and it was found that both of them were potent antibacterial agents against nosocomial bacteria (*Klebsiela pneumoniae*, *Proteus mirabilis*, *Salmonela enterica*, *Enterococcus faecalis*, *Enterococcus epidermis* and *Pseudomonas aeruginosa*). Antibiofouling testing of composite films indicated that the CPDs/PU composite films eradicated almost completely the biofilms of *Pseudomonas aeruginosa* and *Staphylococcus aureus* and about 50% of *Escherichia coli* biofilms.

## 1. Introduction

Bacterial colonization can occur on various surfaces in different public places (hospitals, healthcare facilities, universities, public transport) [1]. A lot of parameters such as shear forces, bacterial motility and electrostatic and hydrodynamic interactions between the surface and microbial cells can contribute to the attachment of pathogens to surfaces [2]. The time that pathogens spend on various surfaces depends on the type of pathogen and can range from a few hours to several days. Besides respiratory, fecal–oral and sexual transmission, pathogen transfer throughout surfaces has a key role in human pathogenic infections, including nosocomial infections [3]. Surfaces present in hospitals (medical equipment surfaces, environment surfaces surrounding patients and corporal surfaces of patients and healthcare workers) may serve both as a reservoir and a vehicle of pathogen transmission [4,5]. The most common surfaces near patients are bedrails and mechanical ventilation tubes for patients who develop ventilator-associated pneumonia [6]. The most common bacteria strains that contaminate hospital surfaces are Methicillin-resistant *Staphylococcus aureus* (*MRSA*), *Pseudomonas aeroginosa*, *Acinetobacter baumannii*, *Escherichia coli* and *Salmonella* spp. [7]. MRSA contamination of environmental surfaces (overbed tables, bedside rails and curtains) in hospital rooms can be transmitted from these surfaces to patients by the hands [8]. Furthermore, it can be found everywhere in hospitals because it is spread by people (patients, visitors or healthcare workers). *Pseudomonas aeruginosa* is a primary cause of ventilated-associated pneumonia in intensive care units [9]. This bacteria is one of the most resistant pathogens to antibiotics. *Acinetobacter baumanii* belongs to the group of pathogens that can adapt to the hospital environment and resist different cleaning and disinfecting agents [10]. The time that pathogens can spend on surfaces determines the risk of pathogen transmission from contaminated surfaces to patients, hospital staff or even visitors [11]. A few parameters affect the ability of pathogens present in hospitals and other healthcare facilities to colonize and survive on surfaces: relative humidity, temperature, ability to form biofilm and the properties of surfaces themselves: wettability, surface roughness and porosity [12].

A biofilm is a microbe assembly enclosed in an extracellular polymeric matrix attached to various surfaces in different environments. A polymeric substance matrix is made up of proteins, polysaccharides or extracellular DNA [13]. The first phase in biofilm formation is the attachment of bacteria to the surface. Further, bacteria proliferate throughout the surface, and at the end, the bacterial community becomes mature. When the biofilm is formed, bacteria that are not resistant to various antibacterial agents can become resistant. This problem of bacterial resistance in applied medicines, i.e., antibiotics, has become more pronounced throughout the years. Due to its big impact on public health, comprehensive research needs to be conducted to understand how to prevent or reduce biofilm formation (which strategy needs to be applied to overcome problems related to biofilm formation).

Up to now, there are a few strategies that can be divided into three groups: (a) changing the properties of susceptible surfaces to prevent biofilm formation, (b) regulating signaling pathways to inhibit biofilm formation and (c) applying external forces to eradicate the biofilm [14]. For example, by using thermal cycling or UV irradiation, surface of potential antibiofilm materials can be treated; various coatings can be deposited on substrates (i.e., trimethylsilane (TMS)/O_2_ and antimicrobial peptides); by using chemical agents that influence quorum sensing, c-di-GMP-, c-di-AMP- and (p)ppGpp-related pathways; to eradicate mature biofilms, external forces (i.e., ultrasound) can be used [14]. 

Photodynamic antimicrobial therapy eradicates pathogens using commercial visible light sources and polymer composites that generate reactive oxygen species [15,16]. It is perfectly safe for medical staff and patients, and there are no toxic compounds that leach from composites and pollute the environment.

In our previous research, we encapsulated carbon quantum dots (CQDs) into polyurethane films and found that these composites eradicated *Stafilococcus aureus* (*S. aureus*) and *Escherichia coli* (*E. coli*) very efficiently for 1 h [17]. Later, we encapsulated carbon polymerized dots (CPDs) into polyurethane films and established their strong antibacterial potential, especially *E. coli*, for 1 h [18]. Other authors encapsulated metal nanoparticles (Cu, Au) into polyurethane films and described their antibacterial activity against *S. aureus* and *E. coli* [19,20,21,22]. Many bactericidal applications of carbon dot composites rely on combining the photodynamic reactive oxygen species production of carbon dots with the antibiotic activity of a transition-metal-based nanoparticle, often containing Ag, Cu, Zn or Ti oxides cores, among others [23]. Fullerenes can inhibit bacterial growth and metabolism. It was established that Gram-positive bacteria are more sensitive to fullerenes due to their higher membrane permeation [24]. The antibacterial activity of fullerenes is based on the electrostatic interaction between fullerenes and bacterial membranes or due to the reactive oxygen generation of fullerenes [25]. 

In this paper, we investigated the structural, chemical, antibacterial, antibiofouling and cytotoxic activities of CPD/polyurethane (CPD/PU) composite films and C_60_/PU composite films. We conducted electrostatic force microscopy (EFM) of composites, which is an electrical mode in atomic force microscopy (AFM) to monitor the variation in samples’ electric fields to obtain information on the charge content and distribution of nanocarbon fillers. We tested both composite film samples on seven different bacterial strains, including *Klebsiela pneumoniae*, *Proteus mirabilis*, *Salmonela enterica*, *Enterococcus faecalis*, *Enterococcus epidermis*, *Shigella flexneri*, *Pseudomonas aeruginosa* and fungi *Aspergilus niger*. Apart from antibacterial activity, we conducted antibiofouling tests against the following bacteria: *Pseudomonas aeruginosa*, *Staphylococcus aureus* and *Escherichia coli.*

## 2. Materials and Methods

### 2.1. Materials

Riboflavin (Carl Roth 97%, Karlsruhe, Germany), ethylenediamine (Carl Roth 99.5%, Karlsruhe, Germany), acetone (Honeywell p.a., Charlotte, NC, USA), toluene (Sigma Aldrich, Rahway, NJ, USA), fullerene C_60_ (Sigma Aldrich, Rahway, NJ, USA), a nylon membrane filter of 100 nm pore size (Tisch Scientific, Cleves, OH, USA), Sensor Green (Invitrogen, Thermo Fischer Scientific, Waltham, MA, USA), D-Chloroform (Sigma Aldrich, Rahway, NJ, USA) were purchased and used as received. Super clear medical grade high stretch polyurethane waterproof transparent TPU film was purchased by DG Xionglin New Materials Technology, Dongguan, China. The thickness of the film was 0.2 mm.

### 2.2. Sample Synthesis

Carbon polymerized dots (CPDs) were synthesized by solvothermal procedure from riboflavin and ethylenediamine mixed in acetone, as described previously [18]. The CPD/PU composite films were synthesized by the following procedure: polyurethane pieces (50 × 50 × 0.2 mm^3^) were inserted in CPD solution in acetone (50 mL). The CPD concentration was 145 mg/mL. To encapsulate CPDs in polyurethane, swelling–shrink encapsulation method was applied [17]. The swelling time was 1 h at room temperature. After that, CPD/PU composite films were dried in vacuum furnace at 80 °C for 12 h.

C_60_ was dissolved in toluene with concentration of 1 g/L. The C_60_/PU composite films were prepared by using the same method as the CPD/PU composite films. The swelling time was 24 h at room temperature.

### 2.3. Sample Characterization

To visualize the surface of CPD nanoparticles, CPD/PU and C_60_/PU composite film AFM was used with AC160 cantilever (MFP-3D Origin, Asylum Research, Oxford Instruments, Santa Barbara, CA, USA). The microscope was operated in tapping and viscoelastic mode at room temperature. Nanoparticle size (diameter and height), typical structure of CPDs and Young’s modulus were calculated from more than 10 AFM images in Gwyddion software (Gwyddion 2.64) [26]. To determine Young’s modulus, microscope was operated in AM-FM Viscoelastic mapping mode (AC 160 cantilever, which operated at 320 kHz and 2 MHz) [27]. Distribution of Young’s modulus was determined from frequency, amplitude and phase of the two modes with a contact mechanics model.

Electrostatic force microscopy (EFM) measurements were performed at room temperature with an MFP 3D Origin (Asylum Research, Oxford Instruments, Santa Barbara, CA, USA). A Si cantilever coated with Ti-Ir (ASYELEC-01) was used. A cantilever resonant frequency v_0_ of 75 KHz, Q factor of 130 and a spring constant k of 2.8 N/m were measured using a thermal noise method. Tip voltage 3 V was typically used. All EFM data were collected from the surface of CPD nanoparticles deposited on freshly cleaved mica by spin coating method and CPD/PU and C_60_/PU composite films. Polymer composite film samples were cleaned with 96% ethanol before each measurement. The distribution of CPDs and C_60_ in the PU was studied by EFM (phase mode).

To determine chemical composition of CPD and fullerene C_60_ film samples, X-ray photoelectron spectroscopy (XPS) analysis of the surface of the CPDs and C_60_ film was carried out on the PHI-TFA XPS spectrometer produced by Physical Electronics Inc. and equipped with Al-monochromatic source and hemispherical electron energy analyzer. The analyzed area was 0.4 mm in diameter, and the analysis depth was 3–5 nm. Pass energy during the XPS analyses was 29 eV, resulting in an energy resolution of 0.65 eV. Two places were analyzed, and XPS spectra were very reproducible. The C 1 s spectrum was aligned to binding energy of 284.5 eV, characteristic of C-C/C-H bonds. The accuracy in the binding energy of XPS spectra was ±0.3 eV. Quantification of surface composition was performed from XPS peak intensities, taking into account relative sensitivity factors provided by instrument manufacturer [28]. XPS data were processed by the Multipak program ver. 9.9. 

Fourier-transform infrared spectroscopy (FTIR) measurements of all samples were performed on a Nicolet iN10 Thermo Fischer Scientific operated in ATR mode. Both C_60_ and CPD samples were deposited on Si substrates using drop-casting method. Spectral resolution was 4 cm^−1^, whereas the spectra were recorded in the range of 400 to 4000 cm^−1^ at room temperature.

UV-Vis spectra of the CPDs, C_60_ toluene solution, CPDs/PU and C_60_/PU composite films were recorded on a Unispec2 LLG spectrophotometer in air at room temperature. The measurement range was from 200 to 700 nm.

### 2.4. Production of Reactive Oxygen Species

To measure singlet oxygen production of composite film samples, we used two methods: measurement of photoluminescence (PL) at 530 nm of sensor green singlet oxygen (SOSG) as fluorescence probe and measurement of decay of absorption spectra of 9,10-Anthracenediyl-bis(methylene)dimalonic acid (ABDA) at 400 nm.

The first method represents the measurement of PL of SOSG, which was used as fluorescence probe. The concentration of SOSG in methanol solution was 12 µmol. The sample (8 × 8 mm^2^) was dipped in methanol solution of SOSG. Both samples, CPDs/PU and C_60_/PU composite films, were irradiated by blue lamp (3 W, V-TAC, Bulgaria) at wavelength of 470 nm. The sample irradiation time ranged from 1 to 11 min, and after irradiation, immediately, the measurements were conducted on Fluoromax + 4 spectrofluorometer (Horiba, Kyoto, Japan). The PL spectra were measured under 488 nm excitation in the range of 500–800 nm [29]. The measurement step was 1 nm.

The second method was based on the measurement of the absorption spectra of 9,10-Anthracenediyl-bis(methylene)dimalonic acid. ABDA (Merck, Burlington, MA, USA) was used as received. A stock solution of ABDA in ethanol (Sigma Aldrich, Sigma Aldrich, Rahway, NJ, USA) of ~10 mM was used. The solution was diluted till absorbance measurement showed that the absorbance at 400 nm was 1 [30]. For irradiation experiments, the solutions were placed into a 10 mm path length cuvette. Then, CPD/PU and C_60_/PU samples (8 × 8 mm^2^) were placed at the bottom of cuvette. Both samples were irradiated by blue light at 470 nm, 3 W. At regular intervals (60, 120 and 180 min), irradiation was stopped, and the absorbance spectra were recorded on an LLG-UNISPEC2 spectrophotometer (LLG, Meckenheim, Germany). All irradiation experiments were repeated three times.

To measure hydroxyl radical production, the following procedure was applied: The samples (CPD/PU and C_60_/PU composite films, 8 × 8 mm^2^) were immersed in 3 mL of terephthalic acid (TA, Sigma Aldrich), water/NaOH (2 × 10^−3^ mol/L) solution. The TA concentration was 5 × 10^−4^ mol/L. Both samples were irradiated with blue lamp at 470 nm in intervals of 0–90 min [31]. The luminescence of the formed hydroxyterephthalic acid (h-TA) solutions was measured on Fluoromax + 4 spectrofluorimeter (Horiba, Kyoto, Japan) under excitation wavelength of 330 nm.

### 2.5. Antibacterial Activity

Antibacterial activity of CPD/PU and C_60_/PU composite films was tested according to International Standard ISO 22196 (Plastics—Measurement of antibacterial activity on plastic surfaces) [32]. Following species of bacteria were used: *Klebsiela pneumoniae ATCC13883* (*K. pneumonie*), *Proteus mirabilis ATCC 14153* (*P. mirabilis*), *Salmonela enterica ATCC 13076* (*S. enterica*), *Enterococcus faecalis ATCC29212* (*E. faecalis*), *Enterococcus epidermis ATCC 12228* (*E. epidermis*), *Shigella flexneri ATCC 12022* (*S. flexneri*), *Pseudomonas aeruginosa ATCC 27853* (*P. aeruginosa*), *Aspergilus niger ATCC 16404* (*A. niger*).

All tests were performed in triplicate, and adequate controls for untreated samples were used (three to measure viable cells immediately and three to measure viable cells after incubation for 24 h). All tested materials—CPD/PU and C_60_/PU composite films—were prepared as flat squares, 50 mm × 50 mm, sterilized by UV lamp at 258 nm for 30 min. Untreated specimens were used for control of bacterial viability, and other material specimens were incubated under blue light at 470 nm for 1 h. Specimens were inoculated with 0.4 mL bacterial suspension of 2.9–15.2 × 10^7^ cell/mL. Test inoculums were covered with films (40 mm × 40 mm), and Petri dishes with test specimens were incubated for 24 h at 35 °C, humidity 90%. Bacteria were recovered with 10 mL of SCDLP, and 10 µL (different dilutions) were plated on LB agar and incubated for 24 h at 35 °C, after which bacterial colonies were counted. The distance from the blue lamp and samples was 50 cm to provide homogeneous irradiation of the samples.

N—number of viable bacteria recovered for test specimen

N = (100 × C × D × V)/A(1)

C—average plate count, D—dilution factor for the plate counted, V—volume (mL) of SCDLP added, A—surface area (mm^2^) of the cover film.

R—antibacterial activity of tested material

R = (Ut − U_0_) − (A_t_ − U_0_) = U_t_ − A_t_ (2)

U_0_—average of log 10 of the number of bacteria from nonirradiated sample in 0 h, U_t_—average log 10 of the number of bacteria from nonirradiated sample after 24 h, A_t_—average log 10 of the irradiated sample after 24 h.

### 2.6. Antibiofilm Activity Testing

For testing antibiofilm effect of composite films, strains of the following bacteria were used: *Staphylococcus aureus* NCTC 6571, *Pseudomonas aeruginosa* ATCC 10332 and *Escherichia coli* NCTC 9001. All materials tested were gently wiped with 70% ethanol, dried and exposed to blue light for 1 h (except untreated control). Using sterile forceps, the materials were then placed in the wells of 6-well microtiter plates. Overnight cultures of different bacteria were diluted 1000-fold (1–5 × 10^8^ cell/mL) and added to 5 mL of fresh, sterile LB medium. Fresh LB medium was added to each well, with a total volume of 5 mL covering the materials. In the test wells, 5 µL of overnight culture was added, while 5 µL of fresh LB medium was added to the control wells. The plate was incubated for 24 h at 37 °C with a rotation rate of 110 RPM.

After incubation, the LB medium was removed, and the materials were washed twice with dH_2_O and dried for 20 min. A 0.1% crystal violet solution was added to each well in a total volume of 3 mL, and the materials were incubated with the stain for 20 min. Following this, the materials were washed twice with dH_2_O and dried. After 20 min, a solution of 30% acetic acid was added to each well in a total volume of 3 mL. Absorbance was measured at 550 nm after 100 µL of acetic acid from each well was transferred to wells of a 96-well microtiter plate. All materials with bacteria were incubated in triplicate, and all controls were incubated in duplicate.

### 2.7. Cytotoxicity

To assess cytotoxicity (antiproliferative activity), standard MTT assays were used, and methods were suitable for materials testing (ISO Standard 10993-5) [33,34]. All tests were performed in triplicate. Human epidermal keratinocyte line (HaCaT) cell line (T0020001) was commercially obtained from AddexBio Technologies (San Diego, CA, USA). These are in vitro spontaneously transformed keratinocytes from histologically normal skin. The HaCaT cells plated in a 96-well flat-bottom plate at a concentration of 1 × 10^4^ cells/well, grown in humidified atmosphere of 95% air and 5% CO_2_ at 37 °C, and maintained as monolayer cultures in RPMI-1640 medium supplemented with 100 µg/mL streptomycin, 100 U/mL penicillin and 10% (*v*/*v*) fetal bovine serum (FBS). Material samples were ethanol washed. Sample extracts were prepared by incubating material samples (1 mg/mL) in RPMI-1640 medium for 72 h at 37 °C. RPMI-1640 medium, in which HaCaT cells were maintained as monolayer cultures, was replaced with undiluted sample extract (100%), 50%, 25% and 12.5% sample extracts (prepared by diluting sample extracts with fresh RPMI-1640). After 48 h of treatment with sample extracts, cell proliferation was determined using MTT reduction assay by measuring absorbance at 540 nm and 670 nm on Tecan Infinite 200 Pro multiplate reader (Tecan Group, Männedorf, Switzerland). The results are presented as a percentage of the control (untreated cells), which was arbitrarily set to 100%.

## 3. Results

### 3.1. Surface Morphology of CPD, CPDs/PU and C_60_/PU Composite Films

**Figure 1:**
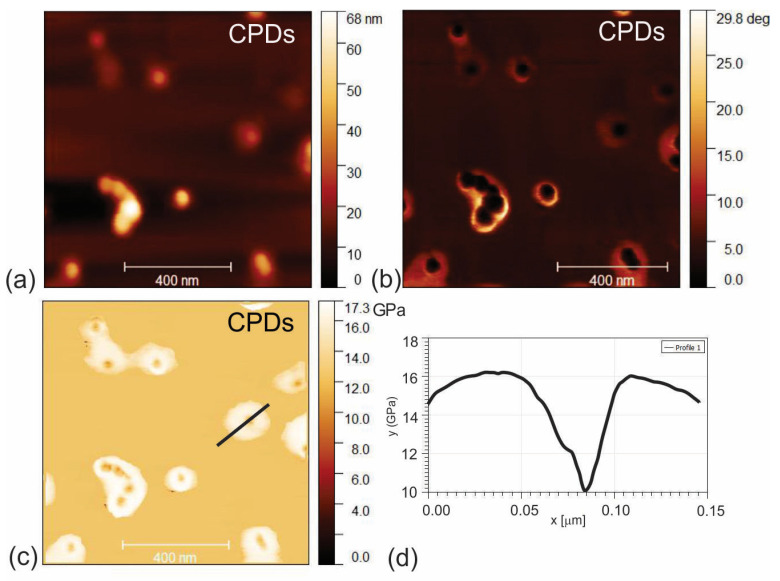
Top view AFM images of CPD nanoparticles: (**a**) height retrace mode, (**b**) phase retrace mode, (**c**) viscoelastic measurement of Young’s modulus and (**d**) profile of Young’s modulus.

The surface morphology of CPD, CPDs/PU and C_60_/PU composite films was investigated by AFM operating in tapping mode. Figure 1a,b show the surface morphology of the CPD nanoparticles recorded in height and phase modes. It was observed that the CPD nanoparticles had a core–shell structure with a typical average core diameter of 33 nm (Figure 1b, phase retrace mode). In our previous research, we established that the average core diameter of CPDs was 35.2 ± 2.1 nm [18]. The average diameter of the whole nanoparticle (core–shell) was 60 nm, whereas the average height was 3 nm.

Viscoelastic measurement of Young’s modulus of the CPDs is presented in Figure 1c,d. It was confirmed that the core–shell structure of the CPDs was revealed by phase measurement. Figure 1d presents Young’s modulus profile of the CPD nanoparticles, designated with a black line in Figure 1c. From Figure 1d, we can see that Young’s modulus of the shell and core was about 16 and 10 Gpa using the Hertz model, respectively.

**Figure 2:**
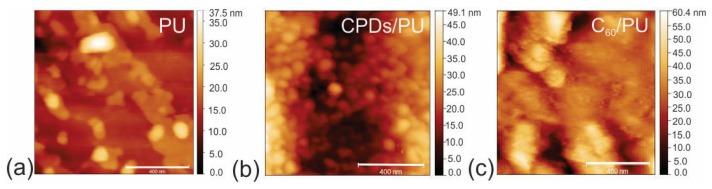
Top view AFM images of neat PU (**a**), CPDs/PU (**b**) and C_60_/PU (**c**) composite films. Scale bar is 400 nm.

Figure 2 shows the surface morphology of neat polyurethane (PU), CPD/PU and C_60_/PU composite films. By Gwyddion software, we calculated the root mean square (RMS) roughness of these polymer composite films. The values of RMS roughness were the following: 3.14 nm (neat PU), 8.60 nm (CPDs/PU) and 8.39 nm (C_60_/PU). Encapsulation of CPDs and C_60_ into the polymer matrix increased RMS roughness by 2.74 and 2.67 times, respectively.

### 3.2. Electrostatic Force Microscopy of CPDs, CPDs/PU and C_60_/PU

Electrostatic force microscopy (EFM) is a very useful method for detecting the charge of a single nanoparticle or nanoparticles dispersed in an insulating polymer matrix or on an insulating surface. This technique enables imaging of the distribution and uniformity of charged carbon-based nanoparticles inside polymers. EFM images can be quantitatively related to charge distributions on a sample surface through mathematical modeling of the tip–sample interaction [35]. Several authors studied the distribution of conductive carbon nanotubes and graphene in a polymer matrix and developed theoretical models that describe the architecture of a polymer composite [36,37,38,39].

**Figure 3:**
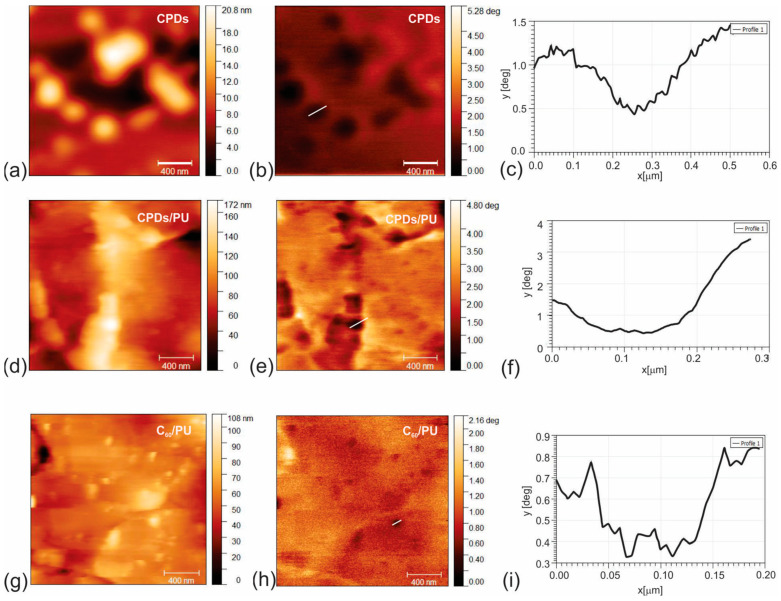
Top view AFM images of surface morphology (left column) and corresponding EFM phase retrace mode images of surface of CPDs (**a**,**b**), CPDs/PU (**d**,**e**) and C_60_/PU composite films (**g**,**h**). Dark voids represent CPDs themselves (**b**), and CPDs in the interior of polymer matrix (**d**) and C_60_ into the polymer matrix (**f**). (**c**,**f**,**i**) show profiles of CPDs, CPDs inside PU and C_60_ in the interior of PU. Tip voltage was 3 V; room humidity was 35%.

All EFM data were collected from the surface of CPD nanoparticles deposited on freshly cleaved mica by the spin coating method and the surface of CPD/PU and C_60_/PU composite films. Figure 3a,d,g show the top view of AFM images (left column) of CPD nanoparticles and CPD/PU and C_60_/PU composite films, respectively. Figure 3b,e,h present the EFM phase images (right column) of CPD nanoparticles and CPD/PU and C_60_/PU composite films, respectively. Data in Figure 3b indicate the presence of negatively charged carriers—electrons in the core of CPDs (negative phase of electrical signal compared with phase on the surface of mica). The presence of electrons in the CPD core helped us to detect CPD nanoparticles inside the polymer matrix (black voids) in Figure 3e. Similar data with smaller intensities were obtained after EFM microscopy of the C_60_/PU composite film. This result indicates that CPD and C_60_ clusters are located in the pores of polyurethane polymer. The intensity of the electrical phase signal was considerably stronger for CPDs/PU than for C_60_/PU, which corresponds to the expected charge distribution. Figure 3c,f,i show the charge distribution profile of CPD nanoparticles, the charge distribution profile of CPD nanoparticles encapsulated in the interior of CPDs/PU and the charge distribution profile of C_60_ clusters in the PU polymer film, respectively. The CPDs have much more electrons (phase in the range of 1–4.5 degrees), see Figure 3e, than the cluster of C_60_ molecules (range 0.3–0.8 degrees), as shown in Figure 3h, located in a nearly identical polyurethane pore.

### 3.3. Chemical Composition

The XPS technique was used to determine the chemical composition of the CPD nanoparticles (elemental composition and characteristic bonds identified) and fullerene C_60_ film. The data presented in Table 1 indicate that the content of C was the highest (83.1 At%), whereas the content of oxygen was the lowest (7.3 At%) for the CPD sample. Table 2 presents the characteristic bonds identified in the CPDs sample and the relative concentration of these bonds in % with respect to the total amount of the specific element (C, O, N). The content of pyrrolic bonds was the highest. C=N-C bonds were also detected, and the presence of these bonds contributed to the new properties of these dots.

Table 1 lists the content of the C_60_ film as well. From this table, we can observe that the content of C 1 s was the highest (90 At%), whereas the content of O 1 s was 10 At% for the C_60_ film sample.

Table 2 lists the binding energies of the characteristic bonds identified in the C_60_ film and the relative concentration of these bonds in % with respect to the total amount of the specific element (C, O). It is known that O_2_ or H_2_O molecules are physisorbed on C_60_ molecules, and the presence of an O 1 s peak in the XPS spectrum of C_60_ originates from that [40].

Figure S1a presents the FTIR spectra of C_60_ (green curve) and the CPD nanoparticles (red curve). The following spectra were identified in the FTIR spectra of the CPD nanoparticles: the peak at 3300 cm^−1^ could be assigned to O-H stretching vibrations in the intermolecular bonding, whereas the peaks at 2956 and 2871 cm^−1^ originated from C-H stretching vibrations of the CH_3_ groups. The peak at 2922 cm^−1^ stemmed from C-H stretching vibrations of the CH_2_ groups. At 1666 cm^−1^, we identified C=N stretching vibrations, whereas at 1435 cm^−1^, we detected O-H bending vibrations. The peak at 1367 cm^−1^ stemmed from N-O symmetric stretching vibrations, whereas the peak at 1290 cm^−1^ was assigned to a C-N stretching aromatic amine. The peak at 881 cm^−1^ originated from C-H bending vibrations [41].

The following peaks were detected in the FTIR spectra of the C_60_ toluene solution: the peak at 3462 cm^−1^ stemmed from O-H stretching vibrations in the intermolecular bonding, the peaks at 2956 and 2860 cm^−1^ were assigned to C-H stretching vibrations of the CH_3_ groups and the peak at 2930 cm^−1^ originated from C-H stretching vibrations of CH_2_ groups. The peak at 1624 cm^−1^ stemmed from C=C stretching vibrations, whereas the peak at 1454 cm^−1^ was assigned to C-H bending vibrations. The peaks at 1256 and 1043 cm^−1^ originated from C-O stretching vibrations. The obtained spectra agree with the recorded XPS spectra.

Figure S1b shows the FTIR spectra of neat PU (black curve), CPDs/PU (red curve) and C_60_/PU (green curve). The following peaks were identified in the spectrum of neat PU: the peak at 3335 cm^−1^ was due to O-H stretching vibrations, the peaks at 2962 and 2866 cm^−1^ stemmed from stretching vibrations of the CH_3_ groups; the peaks at 1738 and 1701 cm^−1^ were assigned to C=O bonds; the peak at 1594 cm^−1^ was due to C=C bonds; the peak at 1525 cm^−1^ originated from N-O stretching vibrations; the peaks at 1414 and 1313 cm^−1^ corresponded to O-H bending vibrations; the peaks at 1228, 1169 and 1073 cm^−1^ were assigned to C-O stretching vibrations; the peaks at 818 and 770 cm^−1^ stemmed from C-H bending vibrations.

In the case of CPD/PU composite films (red curve), we identified the following peaks: the peak at 3206 cm^−1^ was due to O-H stretching vibrations; the peaks at 2956, 2919 and 2850 cm^−1^ stemmed from C-H stretching vibrations; the peaks at 1734 and 1695 cm^−1^ were assigned to C=O bonds, whereas the peak at 1598 cm^−1^ was due to C=C bonds; the peak at 1636 cm^−1^ originated from C=N stretching vibrations and was down-shifted by 30 cm^−1^ compared with the same peak of CPDs nanoparticles; the peak at 1524 cm^−1^ originated from N-O stretching vibrations; the peaks at 1412 and 1313 cm^−1^ were due to O-H bending vibrations; the peak at 1369 cm^−1^ stemmed from N-O symmetric stretching vibrations, and there was an up-shift by 2 cm^−1^ compared with the same peak of CPD nanoparticles; the peaks at 1225, 1108 and 1073 cm^−1^ were assigned to C-O; the peaks at 818 and 770 cm^−1^ could be detected as C-H bending vibrations. The peak at 1290 cm^−1^ identified in the FTIR spectrum of CPD nanoparticles could not be detected in the polymer composite film sample.

The FTIR spectrum of C_60_/PU composite films (green curve) showed the following peaks: the peak at 3302 cm^−1^ was due to O-H stretching vibrations; the peaks at 2919 and 2850 cm^−1^ stemmed from C-H stretching vibrations, whereas the peaks at 1732 and 1698 cm^−1^ were due to C=O bonds; the peak at 1634 cm^−1^ originated from C=C vibrations and was up-shifted by 10 cm^−1^ compared with the same peak of C_60_; the peak at 1525 cm^−1^ originated from N-O stretching vibrations; the peaks at 1412, 1371 and 1312 cm^−1^ originated from O-H bending vibrations; the peaks at 1222, 1105 and 1068 cm^−1^ were due to C-O bonds, whereas the peaks at 813 and 770 cm^−1^ stemmed from C-H bending vibrations.

Table S1 lists the position of the characteristic bonds and shifts (∆) identified in all samples in cm^−1^. From this table, we can observe the position shifts of the characteristic peaks in both composite film samples. The obtained results indicate the encapsulation of certain fillers into the polymer matrix. 

### 3.4. UV-Vis Spectra of CPD and C_60_ Nanoparticles

The UV-Vis absorbance spectra of CPD nanoparticles and C_60_ toluene solution are presented in Figure S2a. Both absorbance spectra were normalized to 1. From this figure, we can observe two peaks only: for the CPD (red curve) acetone solution, we detected peaks at 258 and 330 nm, respectively. The first peak was assigned to the π-π* transition of C=C bonds. The other peak belongs to the n-π* transition of C=O bonds [42]. A similar UV-Vis absorbance spectrum was recorded for the C_60_ toluene solution. Two peaks were identified in the absorption spectrum of C_60_: a peak at 254 nm, which corresponded to C=C due to the π-π* transition, and another peak at 330 nm. 

The UV-Vis absorption spectrum of the CPD/PU composite films (red curve) consisted of a peak at 350 nm and a wide shoulder at 380 nm (Figure S2b). The first peak corresponded to a n–π* transition of C=O, whereas the peak at 380 nm was assigned to C=N-C bonds due to the presence of graphitic nitrogen [43,44]. The absorption spectrum of the C_60_/PU composite films (black curve) shows the presence of only one peak at 392 nm.

### 3.5. Production of Reactive Oxygen Species

#### 3.5.1. Production of Singlet Oxygen

In our previous research, we established that different types of carbon dots (carbon quantum dots (CQDs), graphene quantum dots (GQDs) and doped carbon quantum dots (DCQDs)) produced reactive oxygen species under blue light irradiation [45]. Recently, we found for the first time that CPDs produced singlet oxygen and superoxide anions under blue light irradiation at 470 nm [18]. Until now, there have been two proposed mechanisms of ROS production: Ge et al. claimed that GQDs produce singlet oxygen through energy transfer to molecular oxygen [46], whereas Chong et al. found that besides singlet oxygen, superoxide anion could be involved in the production of ROS [47]. Thus, electron transfer is an intermediate step for the generation of singlet oxygen of GQDs under blue light irradiation.

**Figure 4:**
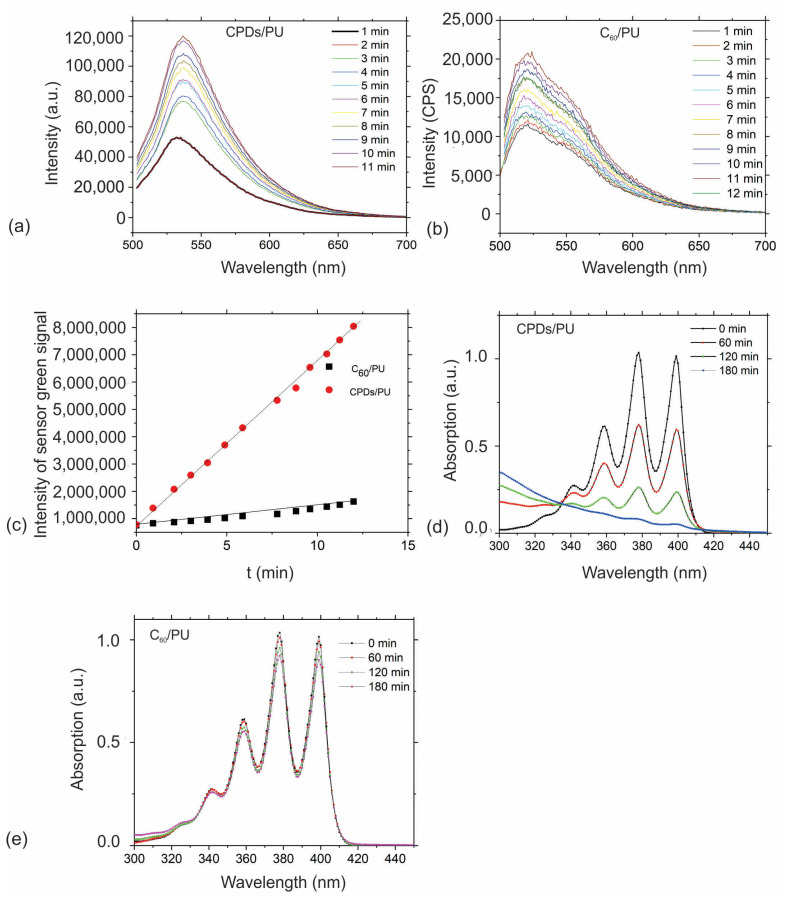
(**a**) Absorption spectra of SOSG used as fluorescence probe at 530 nm in the presence of CPDs/PU samples, (**b**) absorption spectra of SOSG at 533 nm in the presence of C_60_/PU samples, (**c**) production rate of singlet oxygen of CPD/PU and C_60_/PU samples vs. time, (**d**) photobleaching of ABDA in the presence of CPD/PU samples, (**e**) photobleaching of ABDA in the presence of C_60_/PU samples. All absorbance spectra of ABDA were recorded at 398 nm, normalized at the start of the irradiation and averaged over several repeat experiments.

Figure 4 shows the production of singlet oxygen of CPD/PU (Figure 4a) and C_60_/PU (Figure 4b) composite films and the production rate of singlet oxygens of CPD/PU and C_60_/PU samples (Figure 4c). Figure 1a shows that the intensity of the absorption peak of SOSG at 533 nm increased with blue light irradiation time. A similar phenomenon occurred for C_60_/PU samples (Figure 4b). But from Figure 4c, we can observe that the production rate of singlet oxygen is higher for the CPD/PU samples. 

Figure 4d,e represent the decay of the absorption peak of ABDA molecules in the presence of blue-light-irradiated CPD/PU and C_60_/PU samples. From this figure, we can observe the photobleaching of ABDA for different time intervals (60, 120 and 180 min), respectively. As can be seen from Figure 4d,e, the photobleaching of ABDA is much higher for the CPDs/PU samples. In the case of the CPD/PU sample, the decolorization of ABDA was complete after 180 min of blue light irradiation. At the same time, the ABDA decolorization, i.e., the ABDA absorbance intensity, decreased in the presence of C_60_/PU composite films by only 15% after 180 min of blue light irradiation.

#### 3.5.2. Production of OH Radicals

**Figure 5:**
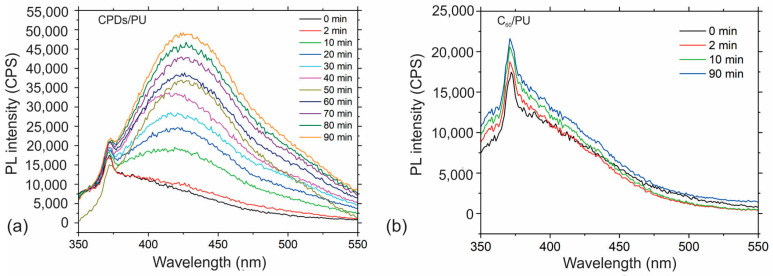
(**a**) PL intensity spectra of h-TA at time intervals of 0–90 min and (**b**) PL intensity spectra of h-TA at time intervals of 0–10 min under excitation of 330 nm. Both samples were irradiated with blue light at 470 nm, 3 W.

It is very important in antibacterial photodynamic treatment to find out if the tested polymer composite films generate other types of ROS. To establish whether these composite films produce hydroxyl radicals, we conducted the measurements using a solution of terephthalic acid as a fluorescent probe. From Figure 5a, we can conclude that there was OH radical production in the CPD/PU samples, whereas there was no hydroxyl radical production in the C_60_/PU samples; see Figure 5b.

### 3.6. Antibacterial Activity of CPD/PU and C_60_/PU Polymer Composite Films

The antibacterial activity of CPD/PU and C_60_/PU composite films was investigated against seven bacteria strains (*K. pneumonie*, *P. mirabilis*, *S. enterica*, *E. faecalis*, *S. epidermis*, *S. flexneri*, *P. aeruginosa*) and fungi (A. niger). Table 3 presents the results of the antibacterial activity of these composites with and without blue light irradiation for 1 h. The results in Table 3 show that the most sensitive bacteria on CPDs/PU were *K. pneumonie*, *P. mirabilis*, *S. enterica*, *E. faecalis*, *E. epidermis* and *P. aeruginosa* under blue light irradiation for 1 h.

The results in Table 3 show that all tested bacteria and fungi were sensitive on the C_60_/PU composite films under blue light irradiation for 1 h. Some bacteria strains are sensitive on the C_60_/PU composite films in the absence of blue light irradiation: *K. pneumonie*, *P. mirabilis*, *S. enterica*, *E. faecalis* and *P. aeruginosa*. Fungi A. *niger* was sensitive on the C_60_/PU composite films in both conditions: with or without blue light irradiation.

The results presented in Table 3 indicate that both types of tested polymer composites are potent antibacterial agents. Under blue light irradiation, the C_60_/PU composite films eradicated Shigella flexneri completely, whereas the CPD composite films eradicated 75% of these bacteria strains. *K. pneumonie* and *S. epidermis* were sensitive on the CPD/PU composite films both with and without blue light irradiation. The C_60_/PU composite films acted very strongly on *K. pneumonie*, *P. mirabilis*, *S. enterica* and *P. aeruginosa*, both with and without blue light irradiation.

The mechanism of their antibacterial activity is the following: both samples generated reactive oxygen species based on the data presented in Figure 4 and Figure 5. The CPD/PU composite films produced singlet oxygen and hydroxyl radicals, whereas C_60_/PU generated only singlet oxygen under blue light irradiation. The produced ROS induced bacterial membrane damage and further oxidative stress [48]. Compared with the results presented in Table 3, we can conclude that C_60_/PU composite films were more toxic on a wide range of tested bacteria.

### 3.7. Antibiofouling Activity of CPDs/PU and C_60_/PU

**Figure 6:**
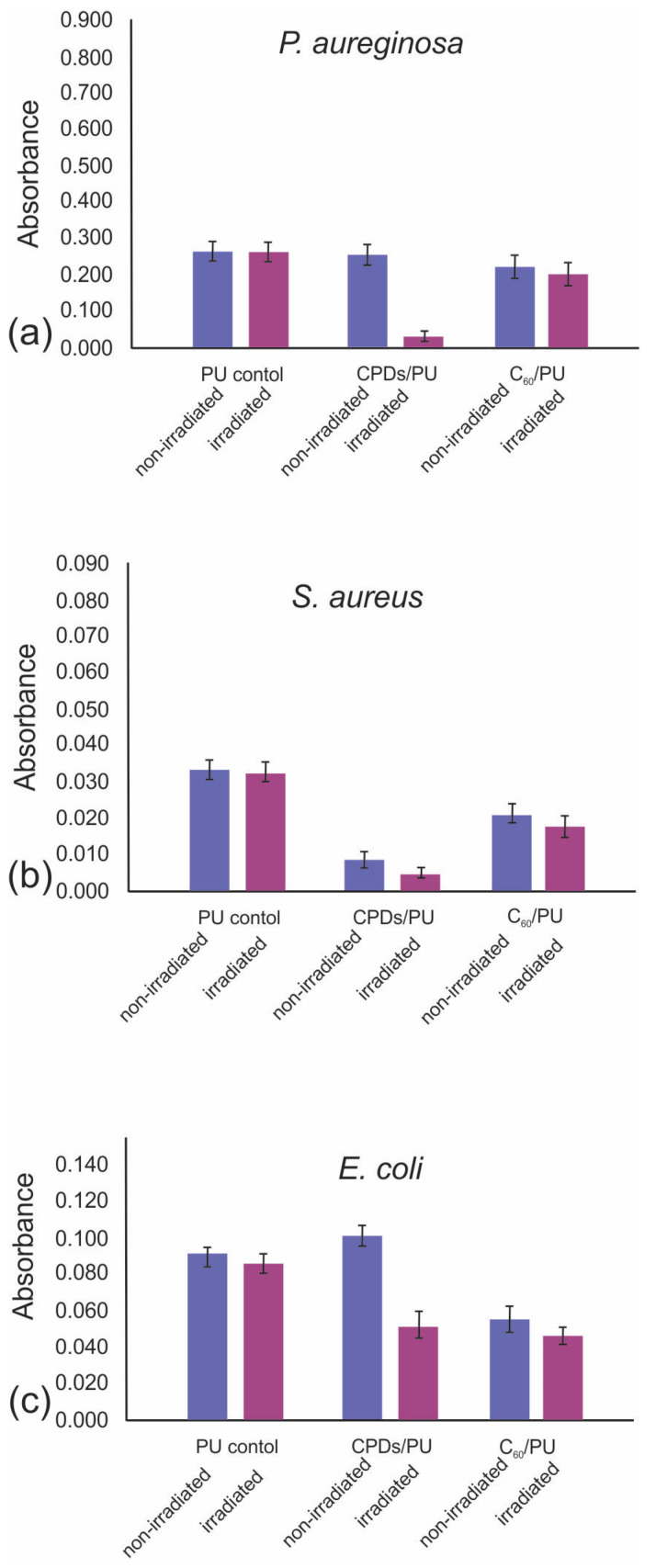
Antibiofouling effect of CPD/PU and C_60_/PU composite films on *P. aureginosa* (**a**), *S. aureus* (**b**) and *E. coli* (**c**) bacterial biofilms with and without blue light irradiation for 1 h. Absorbance is an indicator of the quantity of biofilm formed.

It is very important to observe the formation, reduction or even eradication of biofilms of different bacteria strains. In this research, it was very interesting to observe the antibiofouling activity of the CPD/PU and C_60_/PU composite films against bacteria strains present in hospitals: *P. aeruginosa*, *S. aureus* and *E. coli*. Figure 6a–c shows the antibiofouling activity of both types of testing composite films against three bacteria: *P. aeruginosa*, *S. aureus* and *E. coli*. 

The results indicated the following: biofilm formation on both the CPDs/PU and C_60_/PU composite films was strain dependent, and differences were observed between blue-light-irradiated and nonirradiated samples. Under blue light irradiation, P. aeruginosa biofilm was reduced by 97% on the CPD/PU composite films and by only 20% on the C_60_/PU composite films; see Figure 6a. The CPD/PU composite films had potent antibiofouling activity against *P. aeruginosa* with blue light irradiation. Under the same conditions, *S. aureus* biofilm was reduced by 95% on the CPD/PU composite film and by 65% on the C_60_/PU composite film; see Figure 6b. These composite films showed antibiofilm activity against this bacterium without blue light irradiation: 94% of bacteria biofilm was reduced on the CPD/PU composite films and 75% of bacteria biofilm on the C_60_/PU composite films, respectively. In the case of *E. coli*, CPDs/PU composite films reduced 50% of the bacteria biofilm, whereas the C_60_/PU composite films reduced 45% of the bacteria biofilm under blue light irradiation; see Figure 6c. Both composite films showed better antibiofouling activity against *S. aureus* and *E. coli* under blue light at 470 nm.

### 3.8. Cytotoxicity

To investigate the cytotoxicity of our samples, we tested them on human keratinocyte cell lines (HaCaT). The skin is an organ that is continually self-renewing and participates actively in host defenses [49]. Epidermal cells consist mostly of keratinocytes (about 95%). These cells have an important role as a barrier layer of the epidermis as well as in the initiation and perpetuation of skin inflammatory and immunological responses [50].

But fresh keratinocytes require supplementary growth factors to survive and proliferate in vitro, and once induced to differentiate, they rapidly die and do not allow long-term investigation of differentiation signals [51].

**Figure 7:**
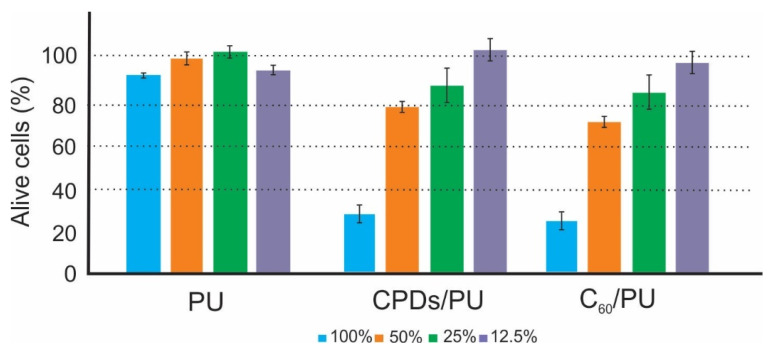
Cytotoxicity of PU (control), CPD/PU and C_60_/PU composite film samples without blue light irradiation determined as a percentage of viable HaCaT cells. The dashed lines highlight the limits of viability according to EN ISO 10993-5: viability >80 corresponds to no cytotoxicity, >60–80 mild cytotoxicity, >40–60 moderate toxicity and <40 severe toxicity.

To minimize these problems, the spontaneously immortalized human keratinocyte cell line HaCaT from adult skin has been proposed as a model for the study of keratinocyte functions. HaCaT is a nontumorigenic monoclonal cell line adapted to long-term growth without feed-layer or supplemented growth factors [51]. Figure 7 shows the viability of HaCaT cells after being treated with neat PU as the control sample and the CPD/PU and C_60_/PU composite film samples.

The control sample did not show any toxicity under any concentration and without blue light irradiation. The CPD/PU composite film sample did not show any toxicity at concentrations of 12.5, 25 or 50% and showed severe toxicity at a concentration of 100%. Similar results were obtained for C_60_/PU composite film samples. The C_60_/PU composite film sample did not show any toxicity at concentrations of 12.5 or 25%, whereas they showed mild toxicity at a concentration of 50% and severe toxicity at a concentration of 100%. All experiments were performed in the dark without irradiation.

## 4. Discussion

In this research, we investigated the antibacterial and antibiofouling activities of CPD/PU and C_60_/PU composite films. Apart from these properties, we investigated their structural, chemical, electrical and optical properties. Structural analysis of nanoparticles encapsulated into polymer films conducted by AFM showed that the CPD nanoparticles had a core–shell structure and an average diameter of 33 nm. Electrostatic force microscopy enabled the determination of the charge type, content and distribution of the CPD nanoparticles and C_60_ clusters and showed that both filler particles were located in the interior of the polymer matrix. It was found that CPDs have much more electrons than the cluster of C_60_ molecules located in the nearly identical polyurethane pore. Before antibacterial and antibiofouling testing, we performed the measurements of ROS production by two methods: (1) by using SOSG as a fluorescence probe and measuring its PL at 530 nm and (2) by using ABDA color and measuring decolorization vs. time. It was established that CPDs/PU and C_60_/PU generated singlet oxygen. Furthermore, it was found later that CPD/PU composite films also produced hydroxyl radicals. None of these composite films showed cytotoxicity against HaCaT cell lines at concentrations ranging from 12.5 to 50%. Antibacterial testing showed that both composite films had strong antibacterial activity against investigated bacteria strains under blue light irradiation for 1 h.

The obtained results indicated that the CPD/PU composite films produced both singlet oxygen and hydroxyl radicals under blue light irradiation at 470 nm. The mechanism of ROS generation is related to negative charge transfer from photosensitizers (CPDs) to oxygen molecules. CPDs are produced from riboflavin and carbon molecules with abundant nitrogen atoms. During the carbonization process in a hydrothermal reactor, nitrogen-doped CPDs are produced. Nitrogen is a rich source of electrons that transfer charge to carbon, which is detected by EFM in the form of an electron cloud over a CPD surface. Similar to an atom, the greatest density of electrons is in the center of CPDs. Since polyurethane is a polymer with the highest oxygen diffusion rate among polymers, oxygens easily collide with CPDs, transferring to one of two detected ROS species and diffusing to the polymer surface [52].

On the other hand, during blue light irradiation of the C_60_/PU samples, only singlet oxygen was generated. Since the C_60_ cluster charge was much smaller than for CPDs, the dominant way for singlet oxygen generation is energy transfer from C_60_ molecules encapsulated in the interior of polymer films to molecular oxygen. C_60_ is composed of pure carbon, and there is no dopant to donate extra electrons. Thus, we prepared two types of composite films with different mechanisms of ROS generation, and it would be interesting to monitor their antibacterial and antibiofouling activities on different bacterial strains.

It is very interesting to emphasize that C_60_/PU composite films eradicated almost completely *S. flexneri* for 1 h. *S. flexneri* is a Gram-negative intracellular pathogen responsible for bacillary dysentery in humans [53]. Per year, more than 1 million deaths occur due to infections with *Shigella* spp. The victims are mostly children from the developing world. The pathogenesis of *Shigella* is based on its ability to invade the colonic epithelium, where it induces severe mucosal inflammation.

We supposed that the antibacterial activity of CPD/PU composite films is predominantly based on ROS generation and further disruption of the bacterial membrane. The C_60_/PU composite films interacted with bacterial membranes by electrostatic forces, and the production of singlet oxygen induced higher membrane permeation compared with the CPD/PU composite films and further bacteria cell death.

Antibiofouling testing showed that the CPD/PU composite films had potent activity against *P. aeruginosa* and *S. aureus* under blue light. The CPD/PU composite films showed better antibiofouling activity against *P. aeruginosa* compared with our previous results related to carbon quantum dots films deposited by the Langmuir–Blodgett method on glass and Si substrates [54]. 

Melanin is a critical component of fungal cell walls, which protect fungi from adverse environmental stress [55]. The results showed that CPDs and C_60_ act differently against *A. niger* due to different charge transfer, C_60_ dissolves in toluene and can encapsulate in the very small pores of polyurethane and at the end, the interaction between the wall of *A. niger* and C_60_ is more intensive than the interaction between CPDs and membrane wall of *A. niger.*

## 5. Conclusions

In this paper, we compared the antibacterial and antibiofouling activities of two types of polymer composite films: CPDs/PU and C_60_/PU. CPDs were synthesized using the solvothermal method using cheap riboflavin powder as a precursor. AFM analysis showed that these dots had a core–shell structure. CPDs and C_60_ were encapsulated into polyurethane films using the swelling–encapsulation–shrink method. EFM analysis indicated that the electronic structure of the CPD/PU and C_60_/PU composite films was related to their ROS generation and antibacterial properties. Both polymer composite films produced ROS: CPD/PU composite films generated both singlet oxygen and hydroxyl radicals, whereas C_60_/PU composite films generated singlet oxygen only. Antibacterial testing showed strong activity of these composite films against seven bacteria strains and one fungus. The antibiofouling activity of the CPD/PU composite films showed almost full eradication of biofilms of *P. aeruginosa* and *S. aureus* under blue light irradiation for 1 h. In this way, both polymer composite films could have a promising future as antibacterial surfaces in hospitals, especially in intensive care and burn units.

## Figures and Tables

**Table 1:** Elements identified in the CPD and C_60_ samples in At%.

	CPD	C_60_
Element	At%	At%
C 1 s	83.1	90.0
O 1 s	7.3	10.0
N 1 s	9.6	0.0

**Table 2:** Characteristic bonds detected in the CPD and C_60_ samples and relative concentration of these bonds %.

Characteristic Bond	Binding Energy (eV)	Relative Concentration (%)	Characteristic Bond	Binding Energy (eV)	Relative Concentration (%)
	CPDs			C_60_	
C 1 s peak C-C/C-H	284.5	80	C 1 s peak C-C/C-H	284.5	79
C 1 s peak C=O	286.5	20	C 1 s peak C-O/C-OH	285.9	20
O 1 s peak C=O	531.4	100	C1 s peak C-O/C-OH	288.7	1
N 1 s peak pyridinic	398.7	45	O 1 s peak O-H	532.2	87
N 1 s peak pyrrolic	399.9	49	O 1 s peak C-O/H_2_O	533.3	13
N 1 s peak C=N-C	396.4	6	-	-	-

**Table 3:** Number of viable bacteria colonies after treatment with the CPD/PU and C_60_/PU composite films in the presence and absence of blue light irradiation.

Bacteria Strains	Irradiated, Incubated N (cell/cm^2^)	Nonirradiated, Incubated N (cell/cm^2^)	R	Irradiated, Incubated N (cell/cm^2^)	Nonirradiated, Incubated N (cell/cm^2^)	R
		CPDs/PU			C_60_/PU	
*K. pneumoniae*	0	0	5.45	0	0	5.45
*P. mirabilis*	0	20 × 10^6^	5.52	0	0	5.52
*S. enterica*	0	5 × 10^7^	4.81	0	2 × 10^7^	4.81
*E. faecalis*	0	10 × 10^5^	5.53	0	6 × 10^5^	5.53
*E. epidermis*	0	0	4.55	0	2 × 10^6^	0.11
*S. flexneri*	64 × 10^2^	82 × 10^4^	2.10	7.6 × 10^3^	9.8 × 10^5^	2.12
*P. aeruginosa*	0	70 × 10^5^	4.95	0	1 × 10^5^	4.95
*A. niger*	8.7 × 10^2^	1 × 10^5^	2.06	0	0	5.47

## Data Availability

The data presented in this study are available on request from the corresponding author.

## Supplementary Materials

The following supporting information can be downloaded at: https://www.mdpi.com/article/10.3390/jfb15030073/s1, Figure S1: (a) FTIR spectra of C_60_ (black curve) and CPDs (red curve) and (b) FTIR spectra of neat PU (black curve), CPDs/PU (red curve) and C_60_/PU (green curve). All spectra are displaced for clarity, Figure S2: (a) UV-Vis spectra of C_60_ (black curve) and CPDs (red curve) and (b) UV-Vis spectra of C_60_/PU (black curve) and CPDs/PU (red curve) composite films. All UV-Vis spectra were normalized to 1. Table S1: Position of characteristic bonds and shifts (Δ) identified in all samples in cm^−1^.
