# Supplementary material for: Antibacterial and Antibiofouling Activities of Carbon Polymerized Dots/Polyurethane and C60/Polyurethane Composite Films
Source: J Funct Biomater. 2024 Mar 17;15(3):73. doi: 10.3390/jfb15030073 (PMC10971164; doi:10.3390/jfb15030073)
Supplement: Supplementary file 1 [file jfb-15-00073-s001.zip › jfb-2889277-supplementary.pdf]

Journal of *Functional Biomaterials*

Supplementary Materials

Antibacterial and Antibiofouling Activities of Carbon Polymerized Dots/Polyurethane and C_{60}/Polyurethane Composite Films

Zoran M. Marković [1,*], Milica D. Budimir Filimonović [1], Dušan D. Milivojević [1], Janez Kovač [2] and Biljana M. Todorović Marković [1,*]

[1] Vinča Institute of Nuclear Sciences, National Institute of the Republic of Serbia, University of Belgrade, Belgrade, Serbia; mickbudimir@gmail.com (M.D.B.F.); dusanm@vinca.rs (D.D.M.)
[2] Jozef Stefan Institute, Department of Surface Engineering, Jamova 39, SI-1000 Ljubljana, Slovenia; janez.kovac@ijs.si
* Correspondence: zoranmarkovic@vin.bg.ac.rs (Z.M.M.); biljatod@vin.bg.ac.rs (B.M.T.M.); Tel.: +381-11-3408582

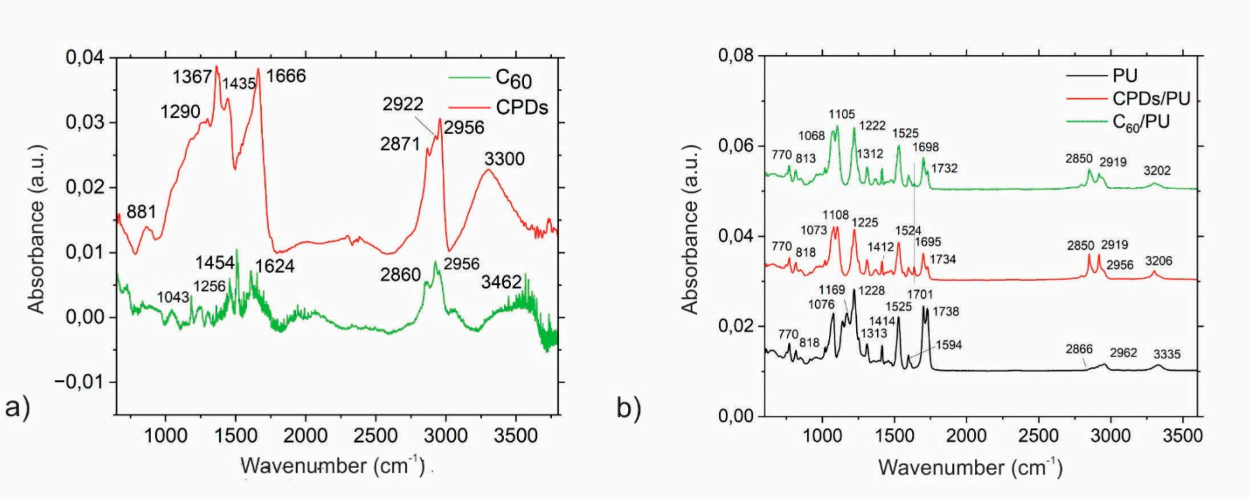

Figure S1. (a) FTIR spectra of C_{60} (black curve) and CPDs (red curve) and (b) FTIR spectra of neat PU (black curve), CPDs/PU (red curve) and C_{60}/PU (green curve). All spectra are displaced for clarity.

Table S1. Position of characteristic bonds and shifts (Δ) identified in all samples in cm^{-1}.

Material	PU (cm^{-1})	CPDs (cm^{-1})	CPDs/PU (cm^{-1})	Δ (cm^{-1})	C_{60} (cm^{-1})	C_{60}/PU (cm^{-1})	Δ (cm^{-1})	Characteristic bonds
	3335	3300	3206	94	3462	3202	-260[1]	O-H
	2962	2956	2956	-6	2956	-	-	C-H stretching vibrations
	-	2922	2919	-3	-	2919	-	C-H stretching vibrations
	2866	2871	2850	-21	2860	2850	-10[2]	C-H stretching vibrations
	1738	-	1734	-4	-	1732	-6	C=O
	1701	-	1695	-6	-	1698	-3	C=O
	-	1666	1636	-30	-	-	-	C=N
	-	-	-	-	1624	1634	10	C=C
	1594	-	1594	0	-	1598	-	C=C
	1525	-	1524	-1	-	1525	-	N-O stretching vibrations
					1454	-	-	C-H bending vibrations
	-	1435	-	-	-	-	-	O-H bending vibrations
	-	1367	1369	+2	-	-	-	N-O stretching vibrations
	1414	-	1412	-2	-	1412	-2	O-H bending vibrations
	1313	-	1312	-1	-	1312	-1	O-H bending vibrations
	-	1290	-	-	-	-	-	C-N
	-	-	-	-	1256	-	-	C-O stretching vibrations
	1228	-	1225	-3	-	1222	6	C-O stretching vibrations
	1169	-	1108	-61	-	1105	64	C-O stretching vibrations
	1076	-	1073	3	1043	1068	25	C-O stretching vibrations
	818	881	818	63	-	813	5	C-H bending vibrations
	770	-	770	-	-	770	-	C-H bending vibrations

[1] (+) determines upshifts related to certain sample;
[2] (-) determines downshifts related to certain sample.

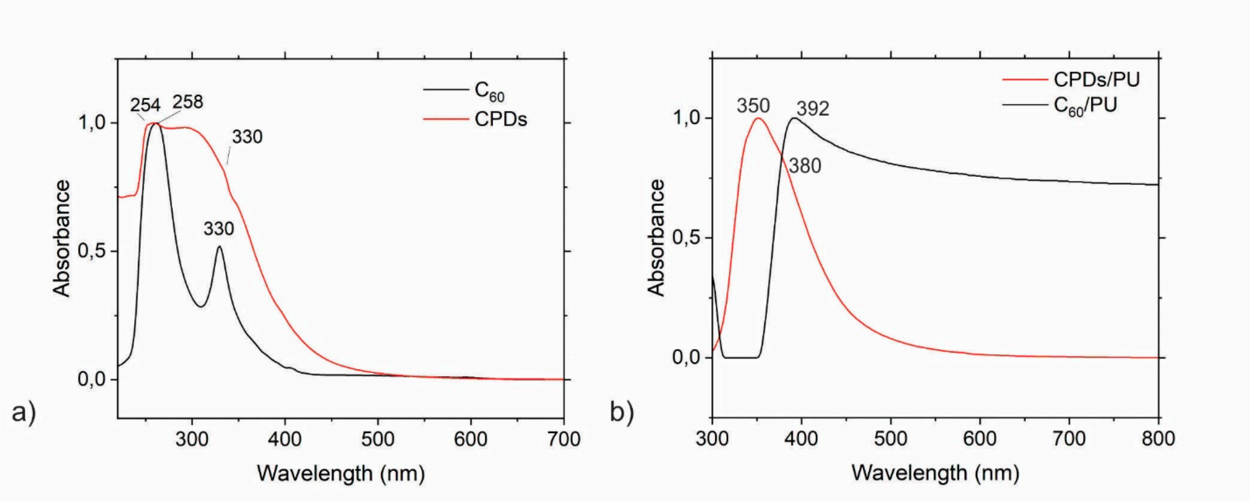

Figure S2. (a) UV-Vis spectra of C_{60} (black curve) and CPDs (red curve) and (b) UV-Vis spectra of C_{60}/PU (black curve) and CPDs/PU (red curve) composite films. All UV-Vis spectra were normalized to 1.
